# Liver injury monitoring, fibrosis staging and inflammation grading using T1rho magnetic resonance imaging: an experimental study in rats with carbon tetrachloride intoxication

**DOI:** 10.1186/s12876-020-1161-3

**Published:** 2020-01-15

**Authors:** Shuangshuang Xie, Hanxiong Qi, Qing Li, Kun Zhang, Longjiang Zhang, Yue Cheng, Wen Shen

**Affiliations:** 1 Department of Radiology, First Central Hospital Clinical Institute, Tianjin Medical University, 22 Qixiangtai Road, Heping District, Tianjin, 300070 China; 2 Department of Radiology, Tianjin First Central Hospital, Tianjin medical imaging institute, 24 Fukang Road, Nankai District, Tianjin, 300192 China; 3 Department of Medical Imaging, Jinling Hospital, Medical School of Nanjing University, 305 Zhongshan East Road, Nanjing, 210002 Jiangsu China

**Keywords:** Liver fibrosis, Inflammation activity, T1rho magnetic resonance imaging

## Abstract

**Background:**

To investigate the merit of T1rho relaxation for the evaluation of liver fibrosis, inflammatory activity, and liver injury monitoring in a carbon tetrachloride (CCl_4_)-induced rat model.

**Methods:**

Model rats from CCl_4_-induced liver fibrosis (fibrosis group: *n* = 41; regression group: *n* = 20) and control (*n* = 11) groups underwent black blood T1rho magnetic resonance (MR) imaging (MRI). Injection of CCl_4_ was done twice weekly for up to 12 weeks in the fibrosis group and for up to 6 weeks in the regression group. MR scanning time points were at baseline and at 2, 4, 6, 8, 10 and 12 weeks after CCl_4_ injection in the fibrosis group and at baseline and at 2, 4, 6 (CCl_4_ withdrawal), 7, 8, 10 and 12 weeks in the regression group.

**Results:**

In the fibrosis group, liver T1rho values increased gradually within week 8 and then decreased. In the regression group, T1rho values dropped gradually after the withdrawal of CCl_4_ and fell below those at baseline. The T1rho values at S0 were lower than those at any other stage (all *P* < 0.05). The T1rho values at G0 were significantly lower than those at any other grade, and G1 was lower than G2 (all *P* < 0.01). The T1rho values mildly correlated with fibrosis stages (*r* = 0.362) and moderately correlated with grades of inflammation (*r* = 0.568). The T1rho values of rats with the same inflammation grades showed no significant difference among different fibrosis stages, and the T1rho values at S3 showed a significant difference among different grades of inflammation (*P* = 0.024). Inflammation grade was an independent variable associated with T1rho values (*P* < 0.001).

**Conclusion:**

T1rho MRI can be used to monitor CCl_4_-induced liver injury, and inflammatory activity had a greater impact on liver T1rho values than fibrosis.

## Background

The main pathological change of liver fibrosis is the accumulation of a large amount of collagen, proteoglycans, and other macromolecules in the extracellular matrix [1]. With regular therapy, liver fibrosis, even early cirrhosis, may regress [2], and early treatment of liver fibrosis can prevent or delay the development of cirrhosis and liver cancer [3–5]. Thus, early and accurate assessment of liver fibrosis are critical for therapeutic decisions and in determining the prognosis. Liver biopsy is the golden standard for the diagnosis and staging of liver fibrosis. However, invasiveness, sampling variability, and poor inter- and intra-observer consistency limit its extensive and repeated use in monitoring disease progression and prognosis [6, 7].

To date, many noninvasive imaging techniques have been developed to evaluate liver fibrosis, such as transient or shear-wave elastography, perfusion or dual energy computed tomography, liver specific contrast-enhanced magnetic resonance imaging (MRI), diffusion weighted MRI, and MR elastography [8–13]. However, these techniques have unique challenges including low repeatability, inaccuracy, radiation exposure, the use of contrast agent, and the requirement of dedicated installation equipment.

T1rho is sensitive to low frequency motional and static processes and can be used to investigate the macromolecular composition and proton exchange in tissues [14–16]. Experimental studies have demonstrated the potential of T1rho MR imaging for monitoring liver injury, diagnosing and staging liver fibrosis, and grading inflammatory activity [17–24]. Furthermore, clinical studies have demonstrated the potential of T1rho MR imaging for detecting and characterizing liver fibrosis, liver cirrhosis, and liver function [25–29]. However, the diagnostic performance of T1rho MR imaging in staging liver fibrosis was different between experimental and clinical studies. Additionally, there a moderate correlation between liver T1rho values and inflammatory activity was demonstrated in a previous rabbit study [18], but no correlation has been observed in human studies [27, 28]. Takayama et al. reported a conflicting result in a human liver study, and indicated that liver T1rho relaxation was not significantly correlated with liver fibrosis [28]. Therefore, more studies are needed to investigate the impact of liver fibrosis and inflammatory activity on liver T1rho values.

Liver fibrosis, depending on its cause, is associated with a number of complicated pathological processes, including steatosis, hepatocellular ballooning, and inflammation [30–33]. Understanding how these processes contribute to elevated T1rho requires careful investigation. In chronic liver disease, the severity of liver fibrosis is usually correlated with a higher inflammatory activity. All previous experimental and clinical studies evaluated the correlation between fibrosis stage or inflammation grades and liver T1rho values separately. In addition, previous animal studies using T1rho MRI to monitor carbon tetrachloride (CCl_4_)-induced liver injury investigated the progression and regression of liver fibrosis in one group, but there was no comparison study between the two states in the same time period. Moreover, after the withdrawal of the CCl_4_ injection, only one time point was evaluated, and this was not compared with the baseline [22, 23]. CCl_4_-induced diffuse liver fibrosis is associated with a greater extent of inflammation, edema, and tissue necrosis. A black blood T1rho MRI has the advantage of decreasing the sensitivity of T1rho quantification to motion [21, 32, 33]. In clinical patients, a liver biopsy is indeed difficult to obtain, while in animal studies, due to free breathing, motion is inevitable. Therefore, the CCl_4_ model using black blood T1rho MRI was selected in this study. The purpose of this study was to comparatively investigate the changes in liver T1rho values with the progression and regression of CCl_4_-induced liver injury, and to evaluate the effect of fibrosis stage and inflammation grade on liver T1rho values.

## Methods

The protocols and procedures were approved by the local Animal Experimentation Ethics Committee (Tianjin First Central Hospital Ethics Committee, 2016N0010KY).

### Animals

In total, 100 adult male Sprague-Dawley rats with a weight of 150–200 g (from China Food and Drug Control Research Institute, Beijing) were used for the experimental study. The animals were housed on a 12-h-light–12-h-dark cycle in an air-conditioned room at 25 °C. Food and water were available ad libitum. Rats were randomly divided into three groups: 58 rats were selected in the liver fibrosis model group, 30 in the liver fibrosis regression group, and 12 in the control group. Rats were acclimatized to standard conditions for 1 week. Liver fibrosis was induced by subcutaneous injection of CCl_4_ twice weekly, with a 40% (v/v) solution of CCl_4_ in corn oil. The first injection dose was 5 ml kg^− 1^ body weight, followed by a dose of 3 ml kg^− 1^ body weight. The rats were weighted twice per week for the purpose of assessing whether drug dose adjustment was required. For baseline measurements, all the rats were subjected to MRI after 1 week of acclimatization. In the fibrosis group, the first injection of CCl_4_ was given to all rats on the same day, and the injection was stopped at the end of week 12. All rats were subjected to MRI at five time points (4, 6, 8, 10, and 12 weeks after the injection), and five to seven rats were randomly selected for sacrifice after MRI for histological examination at each time point. In the regression group, the first injection of CCl4 was given to all rats on the same day. The injection was withdrawn at the end of week 6, and the rats were allowed to recover for 6 weeks. All rats were subjected to MRI at six time points (4 and 6 weeks after the injection, and 1, 2, 4, and 6 weeks after the withdrawal). Five rats were randomly selected for sacrifice (spinal dislocation) after MRI for histological examination at four time points after the withdrawal of CCl_4_ injection (1, 2, 4, and 6 weeks after the withdrawal).

The rats in the fibrosis or progression groups that died or showed severe artifacts in T1rho images were excluded, and alternative rats were randomly selected and supplemented into the group; this was to ensure that a reasonable amount of rats were included in each time point.

In addition, 10 randomly selected rats from the control group were scanned twice to examine scan-rescan reproducibility; the scan interval was 1 week.

### MRI techniques

A 3.0-T MRI instrument (Ingenia 3.0 T TX, Philips Healthcare, Best, Netherlands), equipped with a four-channel phased-array rat coil with a 50-mm diameter (Suzhou, Philips, China), was used for scanning. The rats were administered 0.5% pentobarbital (w/v; 0.6 mL/100 g body weight) anesthesia by intraperitoneal injection (one more injection was given if necessary) before being scanned. To decrease respiratory motion, the rats were placed prone with their heads positioned straight forward. The scans were performed with standard sequences as follows: (A) Coronal T1WI: repetition time (TR) = 600 msec, echo time (TE) =7, field of view (FOV) = 40 mm × 50 mm, matrix = 80 × 96 × 14, slice thickness = 2 mm; (B) axial T2WI: TR = 1141, TE = 60 msec, FOV = 50 mm × 50 mm, matrix = 168 × 156 × 9, slice thickness = 3 mm.

Radio frequency shimming was applied in order to reduce B1 inhomogeneity. Black blood T1rho was determined using a combination of a double inversion recovery (DIR) and 3D balanced turbo field echo (b-TFE) sequence with the following scanning parameters: TR/TE = 5000/9.2 ms, flip angle = 90°, matrix = 168 × 90 × 9, slice thickness = 3 mm, number of slices = 9, FOV = 50 mm × 30 mm, number of signal averaging = 1, spin lock frequency = 500 Hz; echo train length = 1, spin lock time (TSL) = 1, 10, 20, 30, 40, and 50 ms. The total data acquisition time was 9 min and 55 s.

### Image analysis

A T1rho map was generated on a pixel-by-pixel basis on Philips Research Integrated Development Environment software written in Interactive Data Language, using a mono-exponential decay model: M (TSL) = M0 x exp. (−TSL /T1rho), where TSL is the time of the spin-lock pulse.

The data were independently analyzed by one radiologist (SS.X with 7 years experiences in abdominal radiology) who was blinded to the grouping and examination time points of rats. Three representative slices in the upper, middle, and lower liver were selected in order to quantify liver T1rho levels. Four to six ROIs were placed on each axial section of the liver parenchyma region, avoiding potential artifacts and potential high value residual vessel signal. A total of approximately 15 ROIs was obtained for each liver, and the mean value of these 15 ROIs was regarded as the value of the liver T1rho.

In addition, 30 rats randomly selected from the control group, progression group, and the regression group (ten in each group) were independently analyzed by another blinded radiologist in order to examine the inter-reader reproducibility.

### Histopathology

For histological examination, rats were sacrificed on week 2 (*n* = 10), week 4 (*n* = 7), week 6 (*n* = 7), week 8 (*n* = 5), week 10 (*n* = 5) and week 12 (*n* = 7) in the fibrosis group, and week 1 (*n* = 5), week 2 (*n* = 5), week 4 (*n* = 5) and week 6 (*n* = 5) post cessation of the CCl_4_ injection in the regression group. In the control group, 11control rats that were age-matched but without any CCl_4_ insult were killed for histological analysis. Euthanasia was preformed via cervical dislocation. Liver specimens were fixed in 4% phosphate-buffered formaldehyde and embedded in paraffin. Sections 5-μm thick were de-waxed in xylene and rehydrated in ethanol. Standard hematoxylin and eosin (H&E) staining, reticular fiber staining, and Masson staining were used for fibrosis visualization. According to the METAVIR scoring system [34], the stage of liver fibrosis was classified as follows: S0, no fibrosis; S1, enlarged fiber proliferation on portal tracts, and localized perisinusoidal and intralobular fibrosis; S2, peripheral fibrosis in the portal area with the formation of a fiber septa and intact architecture of the liver lobule; S3, fibrous septum accompanied by intralobular structural disorders but without cirrhosis; and S4, definite cirrhosis. The degree of inflammatory activity was graded on a scale of 0–3 (G0 = absent, G1 = mild activity, G2 = moderate activity, G3 = severe activity).

### Statistical analysis

All statistical analyses were performed using SPSS 21.0 (SPSS, Chicago, IL). Data are presented as mean ± standard deviation. The inter-observer agreement and scan-rescan reproducibility were assessed with the intra-class correlation coefficient (ICC). The Mann-Whitney U test was used to compare liver T1rho values at different time points. The Kruskal-Wallis test was used to compare liver T1rho values at different fibrosis stages and inflammation grades. The spearman correlation test and partial correlation test were used to evaluate the correlation between fibrosis stage and inflammation grade with T1rho values. In addition, multiple linear regression analysis was performed to identify factors associated with liver T1rho values. Receiver operative characteristic (ROC) analysis was used to evaluate the diagnostic accuracy of T1rho for significant fibrosis or inflammation. A *P* value < 0.05 was considered statistically significant.

## Results

### Rat characteristics

**Fig. 1:**
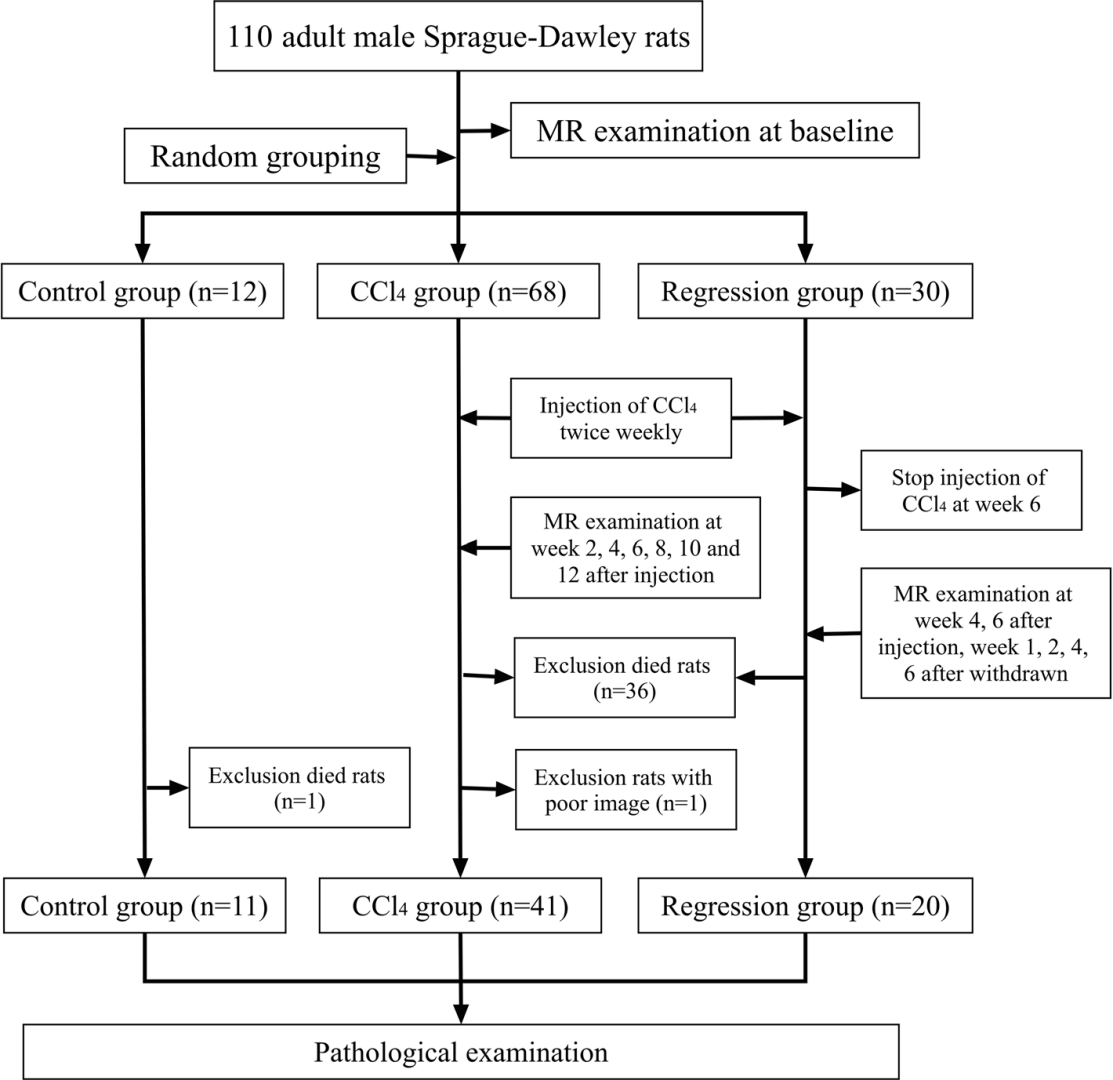
A flow diagram describing the modeling process

The pathology results demonstrated that a small number rats showed fibrosis in stage 1 to 3. As a result, 10 rats were supplemented into the fibrosis group and sacrificed at week 2 after the injection of CCl_4_. In total, 110 rats were used in this study, of which 47 were excluded, including one rat that showed severe artifacts of T1rho imaging in the fibrosis group. Additionally, 26 rats in the fibrosis group, 10 rats in the regression group, and 1 rat in the control group died during the experiment. Finally, 72 rats were included; 41 in the fibrosis group, 20 in the regression group, and 11 in the control group (Fig. 1). In the fibrosis group, the final pathological results showed 2, 14, 4, 6, and 15 rats with S0, S1, S2, S3, and S4, respectively, and 17, 20, and 4 rats with G1, G2, and G3, respectively. In the regression group, the final pathological results showed 2, 5, 2, 3, and 8 rats with S0, S1, S2, S3, and S4, respectively, and 1, 15, 3, and 1 rats with G0, G1, G2, and G3, respectively. In the control group, all rats showed S0 and G0.

### Scan-rescan and inter-reader reliability of the measurements

For the randomly selected ten normal rats, the ICC was 0.9062 (95% confidence interval [CI]: 0.6222–0.9767) for scan-rescan reproducibility. For the randomly selected 30 rats, the ICC was 0.9473 (95% CI: 0.8925–0.9746) for inter-reader T1rho measurement.

### T1rho changes with the progression and regression of liver injury

**Fig. 2:**
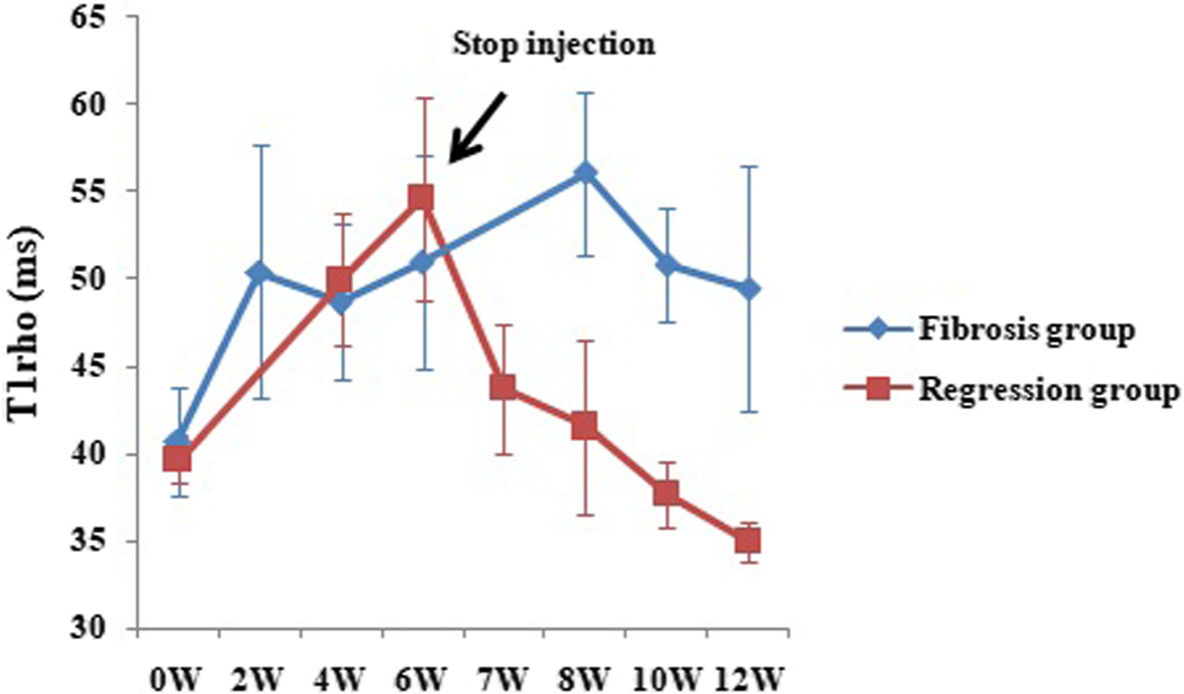
Longitudinal follow-up MR imaging measurement of liver T1rho values. In the fibrosis group, the liver T1rho values gradually increased within week 8, and then dropped gradually from week 8 to 12. In the regression group, the liver T1rho values also increased gradually after the injection of CCl_4_. When the injection stopped at week 6, the liver T1rho values decreased gradually, and even dropped below the baseline values at weeks 10 and 12

In the fibrosis group, the liver T1rho values at all time points (week 2, 4, 6, 8, 10, and 12) were significantly different compared to baseline (all *P* < 0.001). Within week 8, the severity of HF increased with the injection duration of CCl_4_. Correspondingly, we found that there was a trend for T1rho values to increase. Although the T1rho values at week 4 dropped, the difference from the group at week 4 was not insignificantly different compared to week 2 (*P* = 0.643). After week 8, all samples showed stage 4 at week 10 and 12. We found that there was a tendency for T1rho values to decrease, and there was a significant difference between the T1rho values at week 8 and week 10 (*P* = 0.002), but no significant difference between the T1rho values at week 10 and week 12 (*P* = 0.902) (Fig. 2, Table 1).

**Table 1:** Comparisons of liver T1rho values between different time points in fibrosis group

Time of scan	No. of Rats	T1rho range (mean ± SD, ms)	*P*1 value	*P*2 value
baseline	31	35.99–46.85 (40.72 ± 3.08)		
Week 2	10	43.14–64.51 (50.40 ± 7.24)	< 0.001	< 0.001
Week 4	31	41.35–58.16 (48.65 ± 4.46)	< 0.001	0.643
Week 6	24	38.91–69.59 (50.95 ± 6.12)	< 0.001	0.114
Week 8	17	47.77–64.39 (56.09 ± 4.68)	< 0.001	0.004
Week 10	12	45.53–55.66 (50.81 ± 3.23)	< 0.001	0.002
Week 12	7	37.94–56.95 (49.44 ± 7.05)	0.004	0.902

Notes: Time of scan is the time of MRI scan after CCl4 injection. *P*1 and *P*2 values denote the significance level in the Mann-Whitney U test between T1rho values in this time point and T1rho values in baseline and adjacent previous time point, separately

In the regression group, the injection of CCl_4_ was stopped after week 6. Within week 6, the trend of the T1rho values was similar to those of the fibrosis group. After the withdrawal of CCl4, the T1rho values dropped from week 7 to 12, and at week 8, the liver T1rho values dropped to baseline (*P* = 0.521). At weeks 10 and 12, the liver T1rho values were lower significantly than those at baseline (*P* = 0.005, < 0.001), and the T1rho values at week 12 were significantly lower than those at week 10 (*P* = 0.008) (Fig. 2, Table 2).

**Table 2:** Comparisons of liver T1rho values between different time points in regression group

Time of scan	No. of Rats	T1rho range (mean ± SD, ms)	*P*1 value	*P*2 value
baseline	20	37.22–42.11 (39.67 ± 1.39)		
Week 4	20	42.93–55.49 (49.93 ± 3.77)	< 0.001	< 0.001
Week 6	20	47.07–70.24 (54.56 ± 5.87)	< 0.001	0.015
Week 7	20	37.47–55.36 (43.75 ± 3.70)	< 0.001	< 0.001
Week 8	15	35.51–52.36 (41.52 ± 5.04)	0.521	0.033
Week 10	10	34.55–41.11 (37.63 ± 1.87)	0.005	0.012
Week 12	5	33.94–36.23 (34.93 ± 1.15)	< 0.001	0.008

Notes: Time of scan is the time of MRI scan after CCl4 injection. Week 6 is the last time point with CCl4 injection. From week 7 to 12, the CCl4 has been withdrawn. P1 and P2 values denote the significance level in the Mann-Whitney U test between T1rho values in this time point and T1rho values in baseline and adjacent previous time point, separately

### T1rho changes with the progression of fibrosis stage and inflammation grade

All the samples were summarized and the final pathological results showed 15, 19, 6, 9, and 23 rats with S0, S1, S2, S3, and S4, respectively, and 12, 32, 23, and 5 rats with G0, G1, G2, and G3, respectively.

**Fig. 3:**
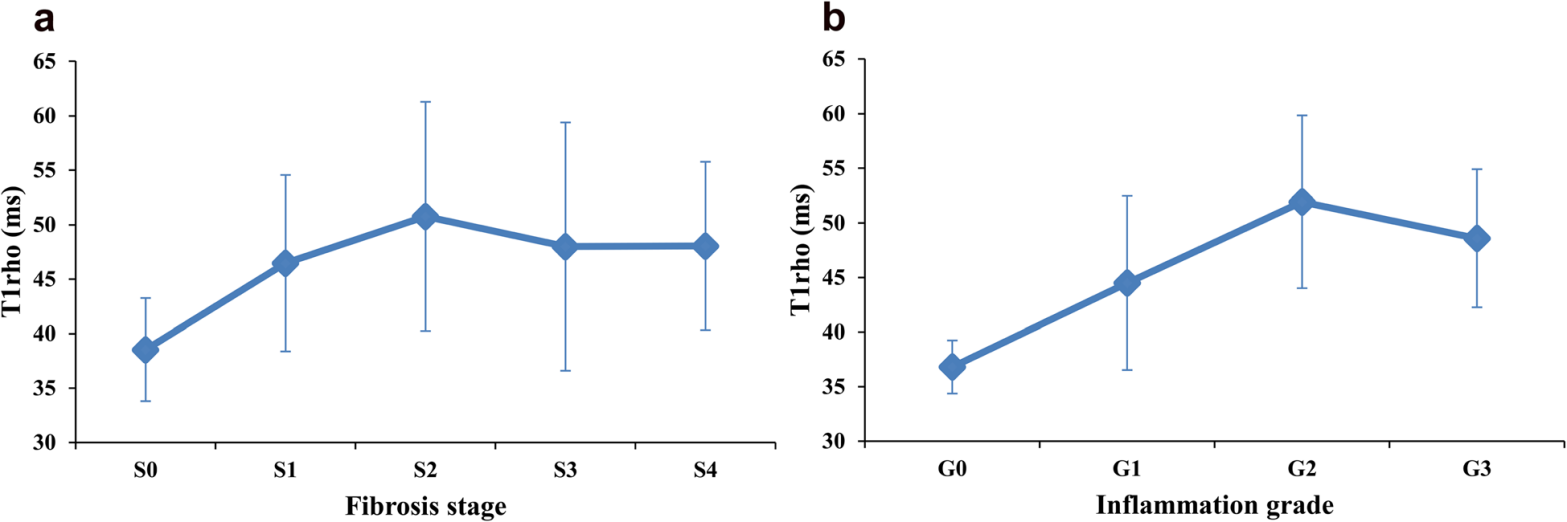
Liver T1rho values of rats at different fibrosis stages (**a**) and inflammation grades (**b**). The liver T1rho values gradually increased from fibrosis stage 0 to 2, but had a decrease at fibrosis stage 3, and no obvious changes at fibrosis stage 4. Liver T1rho values gradually increased from inflammation grade 0 to 2, but had a decrease at inflammation grade 3

**Fig. 4:**
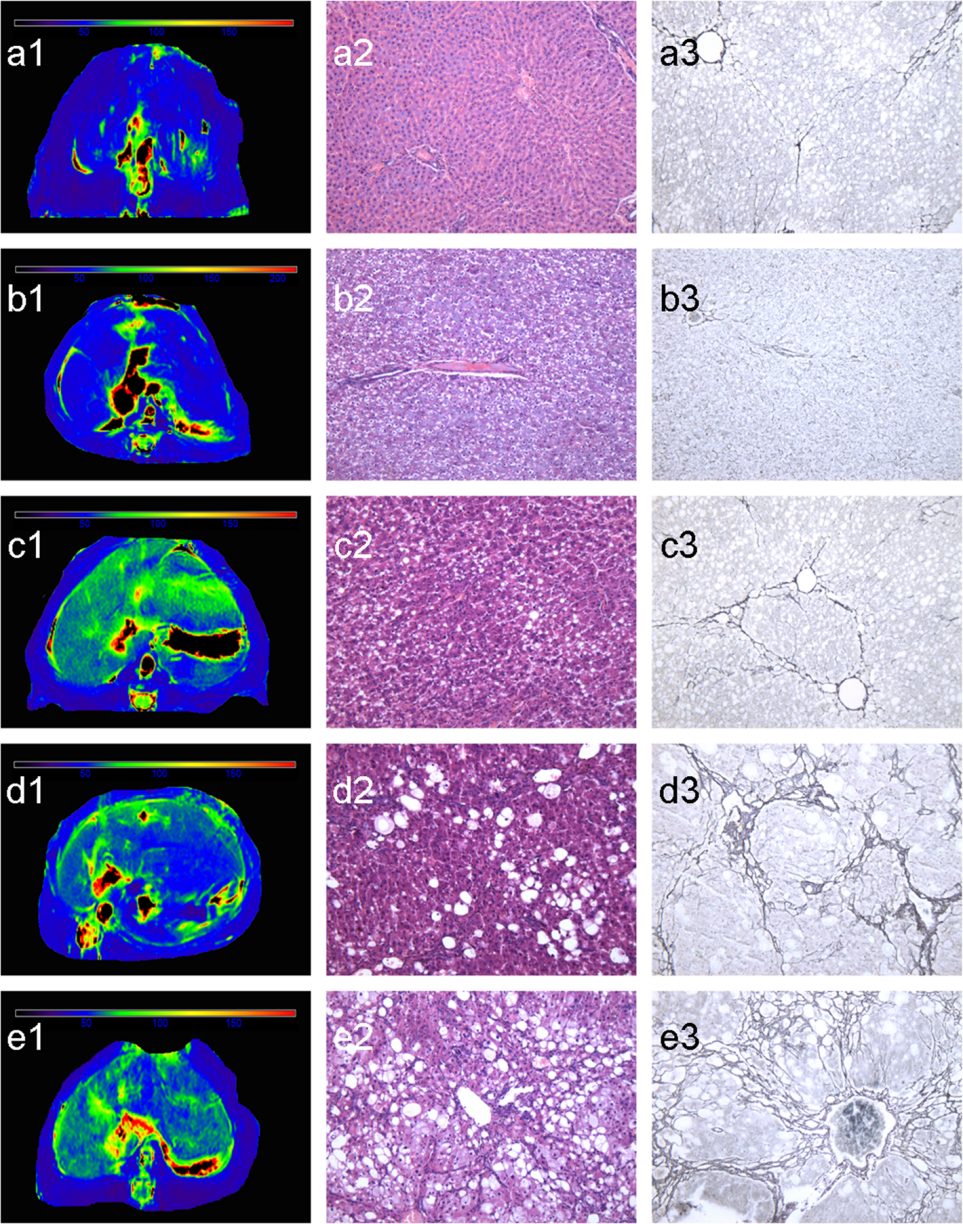
MR T1rho maps, HE, and reticular fiber staining of rat liver at S0 (38.21 ms, a1–3), S1 (46.08 ms, b1–3), S2 (57.51 ms, c1–3), S3 (53.72 ms, d1–3), and S4 (55.89 ms, e1–3). Liver T1rho values increased gradually with the progression of fibrosis; there were decreased at stage 3 (d), and maintained at stage 4 (e)

From fibrosis stage 0 to stage 2, the liver T1rho values increased gradually with the progression of fibrosis; however, they were decreased at stage 3, and maintained at stage 4 (Figs. 3a and 4, Table 3). There was a significant difference between different fibrosis stages (*P* = 0.005), and pairwise comparison showed significant differences between stage 0 and 1 (*P* = 0.008), 0 and 2 (*P* = 0.004), 0 and 3 (*P* = 0.012), and 0 and 4 (*P* = 0.001). However, the liver T1rho values of rats with the same inflammation grades showed no significant difference among different fibrosis stages (G1: *P* = 0.339, G2: *P* = 0.443).

**Table 3:** Liver T1rho values distribution between rats of different fibrosis stage and inflammation grade

Fibrosis stage	No. of rats	T1rho range (mean ± SD, ms)	Inflammation grade	No. of rats	T1rho range (mean ± SD, ms)
S0	15	33.49–52.22 (38.54 ± 4.73)	G0	12	33.49–40.82 (36.81 ± 2.43)
S1	19	33.94–64.51 (46.47 ± 8.09)	G1	32	33.94–64.51 (44.50 ± 7.97)
S2	6	34.22–60.53 (50.78 ± 10.52)	G2	23	37.96–69.59 (51.93 ± 7.92)
S3	9	36.23–69/59 (48.00 ± 11.40)	G3	5	37.94–54.21 (48.58 ± 6.32)
S4	23	34.11–62.51 (48.06 ± 7.71)			

**Fig. 5:**
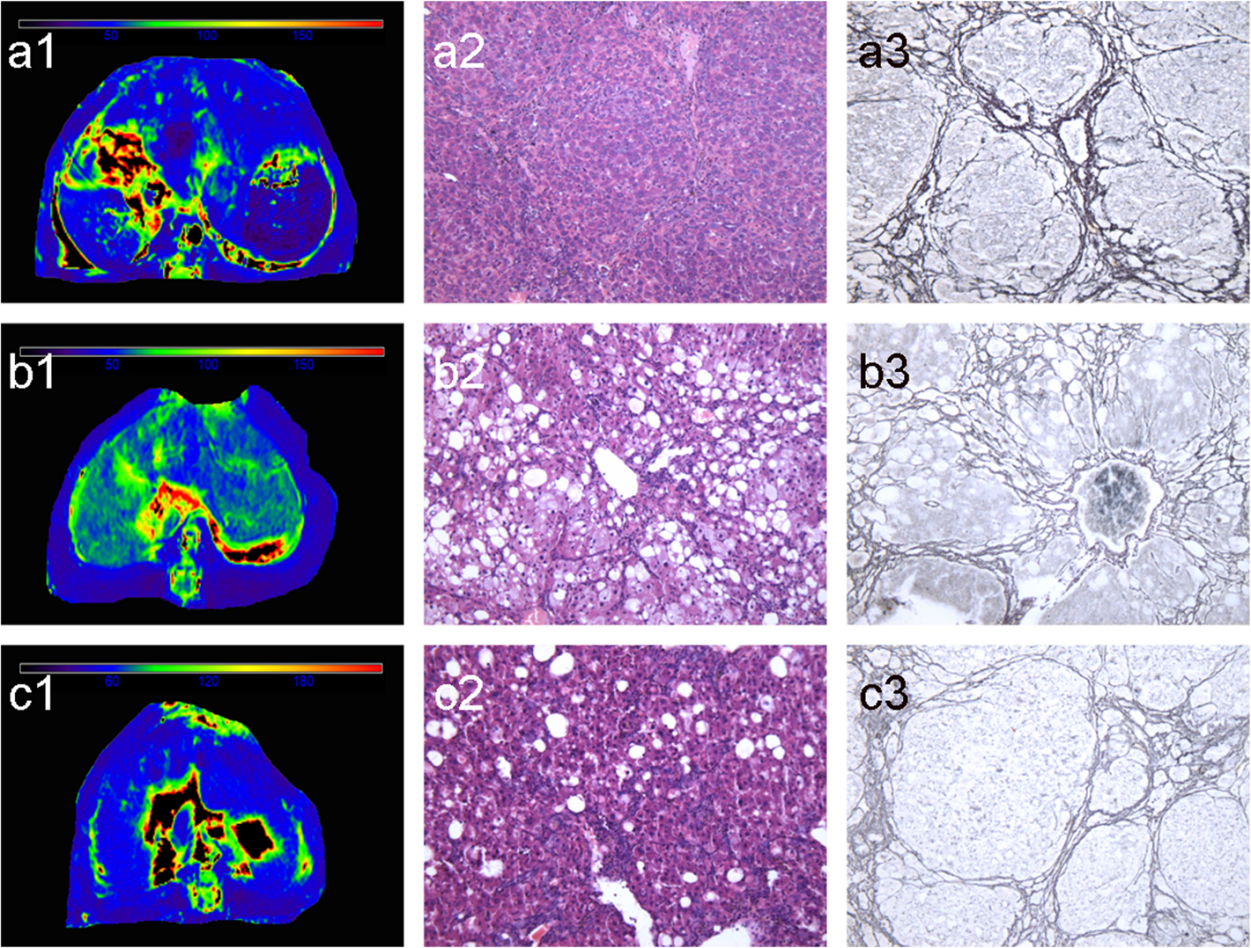
MR T1rho maps, HE, and reticular fiber staining of rat liver with the same fibrosis stage (S4) but different inflammation grade (a1–3: G1&S4, 43.88 ms; b1–3: G2&S4, 55.89 ms; c1–3: G3&S4, 51.76 ms). Liver T1rho values increased from grade 0 to grade 2, but slightly dropped at grade 3

From inflammation grade 0 to grade 2, the liver T1rho values increased with the progression of inflammation; however, these slightly dropped at grade 3 (Figs. 3b and 5, Table 3). Moreover, there was a significant difference between inflammation grades (*P* < 0.001). Pairwise comparison showed significant differences between grade 0 and 1 (*P* = 0.006), grade 0 and 2 (*P* < 0.001), grade 0 and 3 (*P* = 0.009), grade 1 and 2 (*P* = 0.002). However, only the liver T1rho values of rats with fibrosis stage 3 showed a significant difference among the different inflammation grades (S1: *P* = 0.210; S2: *P* = 0.667; S3: G1 = 37.89 ± 1.58, G2 = 53.05 ± 10.72, *P* = 0.024; S4: *P* = 0.050).

### Correlation of liver T1rho values with fibrosis stage and inflammation grade

Liver T1rho showed a low correlation with fibrosis stage (*r* = 0.362, *P* = 0.002) and a moderate correlation with inflammation grade (*r* = 0.568, *P* < 0.001), while fibrosis stage showed a high correlation with inflammation grade (*r* = 0.718, *P* < 0.001). When taking inflammation grade as control variable, there was no significant correlation between liver T1rho values and fibrosis stage (*r* = − 0.086, *P* = 0.476). Additionally, when taking fibrosis stage as control variable, there remained a moderate correlation between liver T1rho values and inflammation grade (*r* = 0.447, *P* < 0.001). In multiple regression analysis, inflammation grade was an independent variable associated with liver T1rho values (b = 6.428, *P* < 0.001, *R*^*2*^ = 0.287).

### Diagnostic accuracy of T1rho for significant fibrosis or inflammation

For the diagnosis of significant liver fibrosis (≥ S2), the AUC was 0.708 (95% confidence interval [CI] = 0.589–0.809). When the optimal cut-off value of 50.7 ms was used, a sensitivity of 53.8% and specificity of 84.8% could be achieved. For the diagnosis of significant inflammation activity (≥ G2), the AUC was 0.819 (95% CI = 0.710–0.900). When the optimal cut-off value of 47.3 ms was used, a sensitivity of 79.3% and specificity of 76.7% could be achieved.

## Discussion

In this study, the ICC for reproducibility of both repeat T1rho MR scan and liver T1rho measurements were higher than 0.9. These data demonstrated the reliability of T1rho MRI, as has been indicated in previous studies [22, 33, 35].

Our results showed that liver T1rho values increased gradually after the injection of CCl_4_ and decreased gradually after withdrawal of CCl_4_ injection. These findings highlight the potential value of T1rho for monitoring liver injury in a CCl_4_-induced fibrosis model, as has been indicated in previous studies [16, 17]. However, in the fibrosis group, we found that the liver T1rho values at week 4 were slightly lower than those at week 2, which was inconsistent with a previous study reported by Zhao et al [23] A possible explanation may be that the liver tissue edema and cellular swelling in our experimental rats was more severe at week 2. This also suggests that it is possible that the degree of liver fibrosis is not the only contributing factor for liver T1rho values, as has been indicated by previous studies [23, 24]. In addition, we found the CCl_4_ was injected continuously after week 8, but liver T1rho values dropped significantly at week 10, and maintained at week 12. We analyzed the rats at weeks 10 and 12, and found that they all showed S4, but the mean liver T1rho values of the S4 rats that appeared after week 8 (week 10 and 12, 49.83 ± 5.58 ms) were lower than those within week 8 (52.88 ± 8.03 ms). This was different to a previous study in a rat biliary duct ligation model that showed a continued increase of liver T1rho values 14 days after cirrhosis occurred [24]. The reason for this difference may be due to the more severe inflammation changes in CCl_4_ model. When we dissected the rat for pathology, the liver volume was first increased and then decreased, especially at week 10 and 12. As a result, the liver T1rho values may also be affected by other factors, such as liver volume and cellular swelling. We analyzed the inflammatory activity of all rat samples at weeks 8 and 10, and found that the rat samples showed inflammation at G1 or G2 at week 8, G2 or G3 at week 10 and 12. The other possible explanation may be due to the increase in tissue necrosis. However, the result may also be affected by other factors, as indicated in a previous study reported by Zhao et al. [23], whereby infiltration of inflammation cells, intracellular deposition of fat vacuoles, degeneration, and necrosis of hepatocytes were consistently observed in livers after CCl_4_ insult. Moreover, in the regression group, the liver T1rho values dropped to normal 2 weeks after CCl_4_ withdrawal (week 8), significantly below normal after 4 weeks (week 10), and continued to fall after 6 weeks (week 12). Furthermore, the pathological results of randomly selected rats at weeks 8 and 10, and all rats at week 12 showed fibrosis in different stages. Only 1 rat showed S0, and all other rats showed S1 to S4. The correlation of fibrosis stage and liver T1rho values was inconsistent with those in the fibrosis group; this also indicated that fibrosis was not the only contributing factor to liver T1rho values. Further careful investigations are needed in order to determine additional contributing factors of liver T1rho values.

Previous clinical and experimental studies have demonstrated the consistent trend of liver fibrosis stages and liver T1rho values [19–25]. In this study, the liver T1rho values at S0 (38.54 ± 4.73 ms) was similar to those described in a previous rat study using the black blood technique (38.38 ± 1.53) [21]. While the liver T1rho values at S1–4 were lower than those in previous rat studies [18, 20], this may be due to the suppression of blood signal by the black blood technique that has been previously demonstrated in clinical patients [32]. When the liver T1rho values were compared among different fibrosis stages, we found that they were significantly lower in the S0 stage compared to the S1, S2, S3, and S4 stages, mildly correlated with liver T1rho values, and had a moderate diagnostic accuracy for predicting significant liver fibrosis (S2–4). These findings highlight the potential value of T1rho MRI for staging liver fibrosis, as has been indicated in previous animal and clinical studies [18, 22, 25, 27]. However, the liver T1rho values at S4 were similar to those of S3 in this study, while the trend in liver T1rho values between different fibrosis stages and the correlation between fibrosis stage and liver T1rho values were inconsistent with previous animal and clinical studies [18–20, 22, 25]. A possible explanation may be due to the large number of rats with S4 from the fibrosis group after week 8, and only eight rats with S4 from the regression group. Because of a factor other than liver fibrosis, the liver T1rho values of these rats were lower than the predicted value. In addition, a previous clinical study with pathological results showed a very high correlation between liver T1rho values and fibrosis stage (r_s_ = 0.99) [25], which was higher than all previous studies, as well as the current study. This may be due to the different causes of liver fibrosis; in previous clinical study, liver fibrosis resulted from virus hepatitis and had a long course of disease, while in animal studies, liver fibrosis mostly resulted from CCl_4_-induced liver injury and had a short course of disease. The different causes may result in different pathological processes.

We also analyzed the difference in liver T1rho values between different inflammation grades and found that liver T1rho values increased gradually from G0 to G2, but were decreased in G3. Significant differences were found between G0 and G1, G2, G3, and G1 and G2, and had a moderate diagnostic accuracy for predicting significant inflammatory activity (G2–3). This indicates the possibility of T1rho MRI in grading inflammation activity. The correlation analysis showed that inflammation grades were moderately correlated with liver T1rho values, which was consistent with a previous animal study [18], but different to previous clinical studies [27, 28]. The differences between clinical and animal studies may also be due to differences in pathological processes that occur as a result of different causes of disease. However, liver fibrosis and inflammation activity usually progress simultaneously in the progress of chronic liver disease. In this study, liver fibrosis was highly correlated with inflammation grade (*r* = 0.718). As a result, we also analyzed the differences in liver T1rho values among fibrosis stages with the same inflammation grade, and among different inflammation grades with the same fibrosis stage. The results showed that the T1rho values at different fibrosis stages with the same inflammation grade were not significantly different, but that the T1rho values at different inflammation grades with fibrosis stage 3 were significantly different (*P* = 0.024). In addition, we found no correlation between fibrosis stage and liver T1rho values when taking inflammation grade as a control variable, but a moderate correlation could be found between inflammation grade and liver T1rho values when taking fibrosis stage as a control variable. Multiple regression analysis showed that inflammation grade was an independent variable associated with liver T1rho values. Thus, the changes in liver T1rho values resulted from the combined effects of liver fibrosis and inflammatory activity, and inflammatory activity had a greater impact on liver T1rho values. When T1rho MRI was used to diagnose liver fibrosis, the impact of inflammatory activity should be considered.

Our study has several limitations. First, a rat model of liver fibrosis may not accurately reflect the pathologic changes of the human liver. Second, the sample size of S2, S3, and G3 was small, and a larger sample size may obviate the statistical error that could have occurred by chance. Third, our study only analyzed the impact of liver fibrosis and inflammatory activity on liver T1rho values, and other pathological changes which may occurred in this model were not considered. Therefore, the conclusive contributing factors of liver T1rho values remain unknown. Future large sample size studies will be needed to develop a conclusive understanding of T1rho changes in relation to liver fibrosis.

## Conclusion

T1rho MRI can be used to monitor liver injury, fibrosis stage, and inflammatory activity induced by CCl_4_. Inflammatory activity had a greater impact on liver T1rho values than fibrosis.

## Data Availability

The datasets used and/or analyzed during the current study are available from the corresponding author on reasonable request.

## Abbreviations

b-TFE: Balanced turbo field echo
DIR: Double inversion recovery
FOV: Field of view
ICC: Intra-class correlation coefficient
MRI: Magnetic resonance imaging
TE: Echo time
TR: Repetition time
TSL: Spin lock time
